# Tuning Sensory Properties of Triazole-Conjugated Spiropyrans: Metal-Ion Selectivity and Paper-Based Colorimetric Detection of Cyanide

**DOI:** 10.3390/s17081816

**Published:** 2017-08-07

**Authors:** Juhyen Lee, Eun Jung Choi, Inwon Kim, Minhe Lee, Chinnadurai Satheeshkumar, Changsik Song

**Affiliations:** 1 Department of Chemistry, Sungkyunkwan University, Suwon, Gyeonggi 16419, Korea; wngusqq@naver.com (J.L.); cej9658@gmail.com (E.J.C.); kiminwon928@gmail.com (I.K.); minhe158@naver.com (M.L.); 2 Graduate School of Nanoscience and Technology, Korea Advanced Institute of Science and Technology (KAIST), Daejeon 34141, Korea; vcsatheeshkumar@gmail.com

**Keywords:** spiropyran, metal ion, cyanide sensing, side-group effect, click reaction

## Abstract

Tuning the sensing properties of spiropyrans (SPs), which are one of the photochromic molecules useful for colorimetric sensing, is important for efficient analysis, but their synthetic modification is not always simple. Herein, we introduce an alkyne-functionalized SP, the modification of which would be easily achieved via Cu-catalyzed azide-alkyne cycloaddition (“click reaction”). The alkyne-SP was conjugated with a bis(triethylene glycol)-benzyl group (EG-BtSP) or a simple benzyl group (BtSP), forming a triazole linkage from the click reaction. The effects of auxiliary groups to SP were tested on metal-ion sensing and cyanide detection. We found that EG-BtSP was more Ca^2+^-sensitive than BtSP in acetonitrile, which were thoroughly examined by a continuous variation method (Job plot) and UV-VIS titrations, followed by non-linear regression analysis. Although both SPs showed similar, selective responses to cyanide in a water/acetonitrile co-solvent, only EG-BtSP showed a dramatic color change when fabricated on paper, highlighting the important contributions of the auxiliary groups.

## 1. Introduction

A range of stimuli (light, temperature, or metal ions) can induce closed forms of well-known photochromic molecules ie, spiropyrans (SPs), to undergo *cis*-*trans* isomerization to give rise to open-ring isomers or merocyanines (MCs) with vastly different physicochemical properties [1]. Such transformations of SPs enable their use as colorimetric sensors due to the vivid colors of the MC forms [2]. SPs are usually modified and functionalized for a certain purpose, such as the development of polymer sensors or dynamic materials, giving selectivity and sensitivity to external stimuli, such as metal ions or temperature. Most SPs are synthesized by condensation between indolenine and benzaldehyde, parts of which are modified to form specific functional groups [3,4]. For example, Shiraishi et al. reported that a coumarin-conjugated spiropyran showed blue fluorescence after nucleophilic addition of CN^−^ under UV light [5]. Stubing et al. compared the absorbance and fluorescence spectra of methyl-1-aza-crown-functionalized SP with different sizes of the aza-crown moiety [6]. These spectra showed the largest changes upon binding of Li^+^ (among alkali metal ions). Perry et al. prepared a pyrene-appended SP receptor for Zn^2+^-selective binding and non-covalent functionalization of carbon surfaces [7]. As described above, evidence from the literature highlights the importance of the addition functional groups to SPs to tailor SP-containing sensors for specific applications. In this respect, the method of easy and high-yielding functionalization to SP needs to be developed. We envisioned that, if an SP has an alkyne moiety [8], the SP can be conjugated with a variety of azide-containing molecules and materials since Cu-catalyzed alkyne-azide cycloaddition (CuAAC) is simple, high-yielding and can tolerate various functional groups [9,10,11]. Therefore, alkyne-containing SPs should have high utilities for developing novel functional materials.

We can also take advantage of the sensory property of the resulting triazole unit of CuAAC in conjunction with that of SPs. Triazole and its derivatives have been used for selective sensing of metal ions [12,13,14,15,16,17]. Thakur et al. synthesized triazole-tethered ferrocene-anthracene conjugates for electrochemical and optical sensing of Pb^2+^ [16]. The compound “turned on” its fluorescence up on the binding of Pb^2+^ ions, and also showed a dramatic change from yellow to a greenish-blue color, allowing naked-eye detection. Kim et al. synthesized a rhodamine triazole-based fluorescent probe for Pt^2+^ detection [12]. A triazole moiety aided selectivity and sensitivity of binding to Pt^2+^ rather than other metal ions in aqueous solution. The synthesized probe also showed a change from colorless to a pinkish-red hue upon binding of Pt^2+^. 

In this study, triazole-conjugated SP molecules are tuned by modification of their side groups. Our strategy was to tune their sensing properties by using a click reaction (CuAAC) between propargyl-functionalized SP and azido molecules. Since sensing in an aqueous environment is important, an ethylene glycol moiety was introduced to SP. This modification renders it more hydrophilic and more sensitive to cyanide in a water environment. We demonstrated that the SP’s sensing properties for metal ions could be easily tuned by click modification. In addition, ethylene glycol-incorporated EG-BtSP, in constrast to simple BtSP, could be utilized as a paper-based colorimetric sensor.

## 2. Materials and Methods 

### 2.1. General

All the chemicals were purchased from Sigma-Aldrich (Seoul, Korea), Alfa Aesar (Seoul, Korea), TCI (Tokyo, Japan), Acros Organics (Geel, Belgium), or Samchun Chemical (Seoul, Korea) and were used as received. ^1^H and ^13^C NMR spectra were recorded using a Bruker 500 MHz spectrometer. The chemical shifts are reported in ppm (δ) with chloroform-*d* (δ 7.26) as an internal standard, and the coupling constants (*J*) are expressed in Hz. UV-VIS absorption measurements were carried out using a UV-1800 (Shimadzu) spectrophotometer. All the metal ions and the anions used in this research are in the form of a perchlorate salt and tetra-*n*-butyl ammonium salt, respectively. High-resolution mass spectra (HRMS) were obtained on a Bruker Daltonics APEX II 3 T FT-ICR-MS. Mass spectra (ESI) were obtained on an Agilent Model:1100 LC-MS mass spectrometers. Column chromatography was carried out using a 100–200 mesh silica gel. Thin layer chromatograph (TLC) analysis was performed on precoated silica gel 60 F254 slides and visualized by UV irradiation. Deuterated solvents for NMR were purchased from Cambridge Isotope Laboratories (Tewksbury, MA, USA).

#### 2.1.1. Synthesis of 3,5-Bis[2-[2-[2-methoxyethoxy]ethoxy]ethoxy] Benzyl Alcohol (1) 

3,5-bis{2-[2-(2-methoxyethoxy)ethoxy]ethoxy}-, methylester [18] (5.68 g, 12.3 mmol) was dissolved in dry tetrahydrofuran (THF) (170 mL) in a 250-mL dropping funnel and this solution was added to LiAlH_4_ (1.20 g, 24.7 mmol) suspended in a dry THF under N_2_ atmosphere. The reaction mixture was stirred under reflux for overnight. The mixture was quenched by adding ice at 0 °C, filtered, and the solvent was then removed under vacuum. The residue was then washed with ethyl acetate (EA) and brine. Finally, the organic phases were dried over anhydrous MgSO_4_, filtered and concentrated to yield **1** (5.36 g, 99%) as a yellow oil. ^1^H (500 MHz, CDCl_3_) δ 6.54 (d, *J* = 2.5 Hz, 2H), 6.41 (t, *J* = 2.5 Hz, 1H), 4.61 (d, *J* = 6 Hz, 2H), 4.11 (t, *J* = 5 Hz, 4H), 3.84 (t, *J* = 5 Hz, 4H), 3.74–3.72 (m, 4H), 3.69–3.64 (m, 8H), 3.56–3.54 (m, 4H), 3.38 (s, 6H). ^13^C (125 MHz, CDCl_3_) δ 160.1, 143.4, 105.5, 100.9, 71.9, 70.8, 70.7, 70.5, 69.7, 67.5, 65.2, 59.0. MS (HRMS): *m/z* calculated for C_21_H_36_O_9_ [M]^+^: 432.2359; found: 432.2361.

#### 2.1.2. Synthesis of 3,5-Bis[2-[2-(2-methoxyethoxy)ethoxy]ethoxy] Benzyl Chloride (2) 

A solution of **1** (1.17 g, 2.70 mmol) in dry dichloromethane (100 mL) was added dropwise to the catalytic amounts of dry dimethylformamide and thionyl chloride (0.45 g, 3.79 mmol) at 0 °C. After stirring at room temperature for 10 h, unreacted thionyl chloride and dichloromethane were removed under reduced pressure and extracted with ethyl acetate. The combined organic extracts were dried over Na_2_SO_4_, filtered, and evaporated under vacuum. The product was concentrated as a pale-yellow oil **2** (0.94 g, 77%). ^1^H (500 MHz, CDCl_3_) δ 6.54 (d, *J* = 2.0 Hz, 2H), 6.44 (d, *J* = 2.0 Hz, 1H), 4.49 (s, 2H), 4.11 (t, *J* = 5.0 Hz, 4H), 3.84 (t, *J* = 5.0 Hz, 4H), 3.74–3.72 (m, 4H), 3.69–3.65 (m, 8H), 3.56–3.54 (m, 4H), 3.38 (s, 6H). ^13^C (125 MHz, CDCl_3_) δ 160.0, 139.4, 107.4, 101.6, 71.9, 70.8, 70.7, 70.6, 69.6, 67.6, 59.1, 46.3. MS (HRMS): *m/z* calculated for C_21_H_35_ClO_8_ [M]^+^: 450.2020; found: 450.2022.

#### 2.1.3. Synthesis of 3,5-Bis[2-[2-(2-methoxyethoxy)ethoxy]ethoxy] Benzyl Azide (3)

A solution of compound **2** (0.89 g, 2.00 mmol), NaN_3_ (0.52 g, 8.00 mmol) in dry dimethylformamide (10.0 mL) were stirred at 60 °C for 24 h. The reactant was then cooled to room temperature and quenched by addition of water. The mixture was evaporated under reduced pressure, and the residue was added ethyl acetate and washed with water and brine. The organic layer was dried over Na_2_SO_4_, filtered, and concentrated. Compound **3** was obtained as a pale-yellow liquid (0.70 g, 77%). ^1^H (500 MHz, CDCl_3_) δ 6.47–6.45 (m, 3H), 4.24 (s, 2H), 4.12–4.10 (m, 4H), 3.86–3.84 (m, 4H), 3.75–3.72 (m, 4H), 3.70–3.67 (m, 4H), 3.67–3.65 (m, 4H), 3.56–3.54 (m, 4H), 3.38 (s, 6H). ^13^C (125 MHz, CDCl_3_) δ 160.2, 137.5, 107.0, 101.4, 72.0, 70.9, 70.7, 69.7, 59.1, 31.0. MS (HRMS): *m/z* calculated for C_21_H_35_N_3_O_8_ [M]^+^: 457.2424; found: 457.2422.

#### 2.1.4. Synthesis of 8-Methoxy-3′,3′-dimethyl-6-nitro-1′-(prop-2-yn-1-yl)spiro[chromene-2,2′-indoline] (6)

A mixture of 3,3-dimethyl-2-methylene-1-(prop-2-yn-1-yl)indoline **4** (3.91 g, 125 mmol) was added to 2-hydroxy-3-methoxy-5-nitrobenzaldehyde **5** (2.70 g, 137 mmol) in ethanol (30.0 mL), and sonicated for two hours. The residue was then evaporated and diluted in ethyl acetate. The organic layer was washed with water and brine and dried over Na_2_SO_4_. After evaporation, the crude mixture was purified by column chromatography and obtained compound **6** (2.81 g, 60%). ^1^H (500 MHz, CDCl_3_) δ 7.70 (d, *J* = 2.5 Hz, 1H), 7.62 (d, *J* = 2.5 Hz, 1H), 7.20–7.23 (td, *J* =7.5 Hz, 1H), 7.09–7.11 (dd, *J* = 7.3 Hz, 1H), 6.90–6.93 (m 2H), 6.81 (d, *J* = 7.5 Hz, 1H), 5.89 (d, *J* = 10 Hz, 1H), 4.04 (dd, J = 18 Hz, 2.5 Hz, 1H), 3.86 (dd, J = 18.5 Hz, 2.5 Hz, 1H), 3.76 (s, 3H), 2.10 (t, 1H), 1.23 (s, 3H), 1.12 (s, 3H). ^13^C (125 MHz, CDCl_3_) δ 149.1, 147.5, 145.8, 140.6, 136.1, 128.7, 127.7, 121.7, 121.3, 120.2, 118.2, 115.4, 108.0, 107.9, 105.8, 79.7, 71.4, 56.3, 52.5, 32.6, 26.0, 20.0. MS (HRMS): *m/z* calculated for C_22_H_20_N_2_O_4_ [M]^+^: 376.1423; found: 376.1418.

#### 2.1.5. Synthesis of EG-BtSP

A solution of compound **6** (0.15 g, 0.39 mmol), compound **3** (0.18 g, 0.39 mmol), CuSO_4_·H_2_O (5 mol %), and sodium ascorbate (10 mol %) in a mixture of THF-H_2_O (1:1 *v*/*v*) was stirred for 12 h at room temperature. The residue was evaporated under vacuum, dissolved in chloroform and washed with water and brine. The organic layer was dried over Na_2_SO_4_ and concentrated to afford the crude product. The product (0.19 g, 56%) was purified by column chromatography (silica gel; eluent, Hexane: EA = 1:10). ^1^H (500 MHz, CDCl_3_) δ 7.67 (d, *J* = 2.5 Hz, 1H), 7.54 (d, *J* = 2.5 Hz, 1H), 7.44 (s, 1H), 7.04–7.08 (m, 2H), 6.88 (d, *J* = 10.5 Hz, 1H), 6.82–6.85 (m, 1H), 6.41–6.40 (m, 2H), 6.23 (d, *J* = 2 Hz, 2H), 5.86 (d, *J* = 10 Hz, 1H), 5.36 (d, *J* = 15.5 Hz, 1H), 5.29 (d, *J* = 15 Hz, 1H), 4.71 (d, *J* = 17 Hz, 1H), 4.58 (d, *J* = 17 Hz, 1H), 3.95–3.99 (m, 3H), 3.79 (t, *J* = 5 Hz, 4H), 3.70–3.72 (m, 4H), 3.64–3.68 (m, 12H), 3.53–3.55 (m, 4H), 3.37 (s, 6H), 1.29 (s, 3H), 1.18 (s, 3H). ^13^C (125 MHz, CDCl_3_) δ 160.3, 148.7, 147.0, 145.7, 145.6, 140.5, 137.0, 135.8, 128.6, 127.5, 122.7, 121.9, 121.7, 119.6, 118.2, 115.5, 107.8, 107.7, 106.4, 106.2, 101.1, 71.9, 70.8, 70.6, 70.5, 69.5, 67.5, 59.0, 56.1, 53.9, 53.0, 39.6, 26.3, 19.9. MS (ESI): *m/z* calculated for C_43_H_56_N_5_O_12_ [M + H]^+^: 834.93; found: 834.47.

#### 2.1.6. Synthesis of BtSP 

A solution of compound **6** (0.16 g, 0.43 mmol), benzyl azide (0.06 g, 0.43 mmol), CuSO_4_·H_2_O (5 mol %), and sodium ascorbate (10 mol %) in a mixture of THF-H_2_O (1:1 *v*/*v*) was stirred for 12 h at room temperature. The residue was evaporated under vacuum, dissolved in chloroform and washed with water and brine. The organic layer was dried over Na_2_SO_4_ and concentrated to yield the crude product. The product was purified by column chromatography (silica gel; eluent, Hexane: EA = 3:1; 0.10 g, 48%). ^1^H (500 MHz, CDCl_3_) δ 7.66 (d, *J* = 2.5 Hz, 1H), 7.51 (d, *J* = 2.5 Hz, 1H), 7.46 (s, 1H), 7.29–7.30 (m, 3H), 7.04–7.08 (m, 4H), 6.83–6.87 (m, 2H), 6.38 (d, *J* = 7.5 Hz, 1H), 5.85 (d, *J* = 10 Hz, 1H), 5.47 (d, *J* = 15.5 Hz, 1H), 5.39 (d, *J* = 15.5 Hz, 1H), 4.72 (d, *J* = 17 Hz, 1H), 4.58 (d, *J* = 16.5 Hz, 1H), 3.60 (s, 3H), 1.29 (s, 3H), 1.18 (s, 3H). ^13^C (125 MHz, CDCl_3_) δ 218.3, 148.7, 147.0, 145.7, 145.6, 140.5, 135.9, 135.1, 129.0, 128.6, 128.5, 127.6, 127.3, 122.7, 121.9, 121.7, 119.6, 118.3, 115.5, 107.8, 107.7, 106.4, 56.1, 53.9, 53.0, 39.6, 26.3, 19.9. MS (HRMS): *m/z* calculated for C_29_H_27_N_5_O_4_ [M]^+^: 509.2063; found: 509.2062.

### 2.2. UV-VIS Spectrum Measurement for Absorption Spectra

#### 2.2.1. Metal Screening of EG-BtSP and BtSP

Separate EG-BtSP (c = 5 × 10^−5^ M), BtSP (c = 1 × 10^−4^ M) and metal (c = 1 × 10^−2^ M) stock solutions (perchlorate salts in acetonitrile) were prepared for UV-VIS measurements. UV-VIS absorption spectra were obtained with SP solutions (2 mL) 30 min after the addition of 1 equiv. of each metal ion solution.

#### 2.2.2. Dielectric Constant

EG-BtSP and BtSP compounds (c = 5 × 10^−5^ M and 1 × 10^−4^ M, respectively) were prepared in nine solvent mixtures using water/acetonitrile (total 2 mL), varying the water composition up to 90% water (*v*/*v*) in acetonitrile. After 30 min, UV-VIS absorption analysis was performed on the soluble samples. 

#### 2.2.3. Anion Screening of EG-BtSP and BtSP

For UV-VIS anion screening, the stock solutions of EG-BtSP and BtSP (c = 2 × 10^−5^ M) were prepared in a water/CH_3_CN mixture (9/1 and 1/1 *v*/*v*, respectively) along with each of anion solution (c = 5 × 10^−2^ M) as a *n*-Bu_4_N^+^ salt. UV-VIS spectrophotometric measurements were performed with SP solutions (2 mL) 30 min after addition of each anion solution (40 μL, about 50 equiv.).

#### 2.2.4. Paper-Based Sensor Test of EG-BtSP and BtSP

For the paper sensor test, we prepared a 10^−1^ M SP stock solution in acetonitrile, and filter paper cuts (1 cm × 1 cm) were coated with this solution via a dipping method. After drying for 24 h, the SP-loaded paper was dipped into aqueous cyanide solutions at different concentrations (1 mM, 10 mM, 20 mM, 50 mM, 100 mM, and 500 mM). The paper was then dried in air at room temperature.

## 3. Results and Discussion

**Scheme 1:**
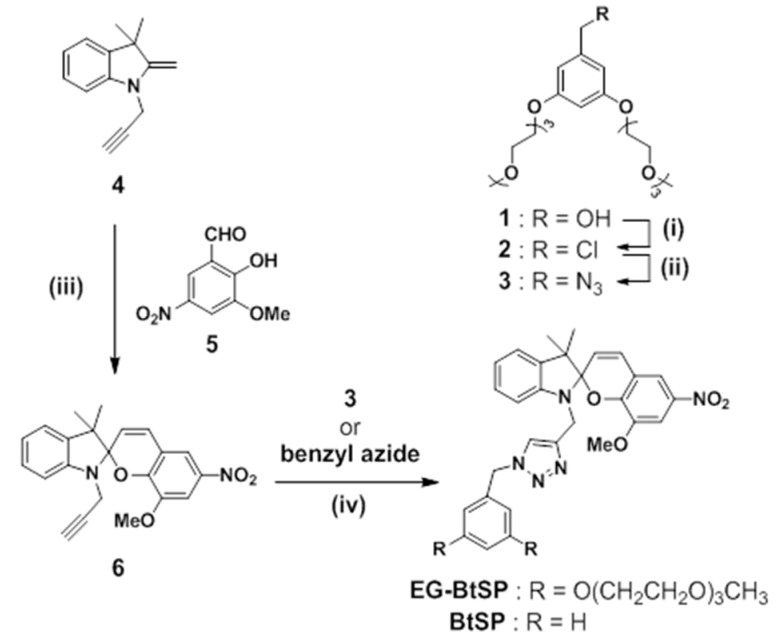
Synthesis of EG-BtSP and BtSP from an Azide-Functionalized Spiropyran. (i) SOCl_2_, DMF, DCM, room temperature, 10 h, 77%; (ii) NaN_3_, DMF, 60 °C, 24 h, 85%; (iii) EtOH, sonication, 2 h, 60%; (iv) CuSO_4_·5H_2_O (5 mol %), sodium ascorbate (10 mol %), THF/H_2_O (1:1 *v*/*v*), room temperature, 12 h, 56% (EG-BtSP) and 48% (BtSP).

Since azide-containing molecules can be easily conjugated with alkyne-functionalized SP via Cu-catalyzed azide-alkyne cycloaddition (CuAAC), bis(triethylene glycol)-attached benzyl azide **3** was prepared from the corresponding alcohol **1** via reduction [19], chlorination [20], and nucleophilic substitution [21] (Scheme 1). The propargyl-functionalized SP **6** was synthesized by condensation between propargyl indole **4** and 2-hydroxy-3-methoxy-5-nitrobenzaldehyde **5** according to a published method [8]. The CuAAC of propargyl-SP **6** with azide **3** gave rise to a good yield (56%) of a bis(triethylene glycol)-functionalized SP with a triazole linkage EG-BtSP, as illustrated in Scheme 1. BtSP, which does not have ethylene glycol moieties, was also prepared by a similar method. Our hypothesis was that addition of ethylene glycol moieties would render SPs hydrophilic, enhancing their sensing abilities in aqueous environments.

The photochromic behaviors of EG-BtSP and BtSP were investigated in acetonitrile. The initial solutions of EG-BtSP and BtSP in acetonitrile (~0.10 mM) were colorless. When 365-nm UV light was illuminated on the solutions of SP molecules, the color of both solutions changed to blue, showing the same new absorption peak at around 600 nm. UV irradiation caused the C-O bond cleavage of SP molecules, resulting in an open MC form, as also seen in evidence from the literature [22] The spontaneous reverse isomerization from MC to SP occurred under visible light. Understandably, this result showed that the photochromic property of EG-BtSP and BtSP comes from the SP moiety, not from the auxiliary chains.

**Figure 1:**
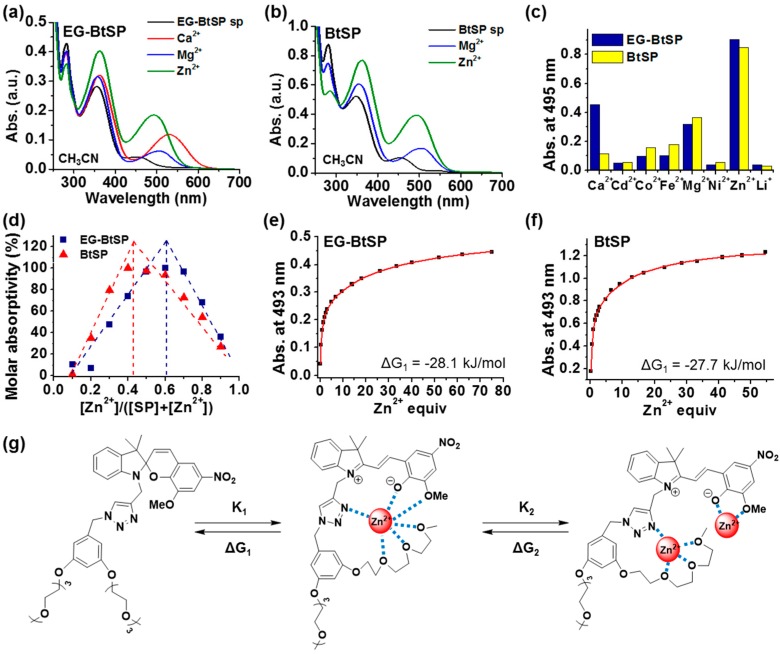
(**a**,**b**) Absorption spectra of EG-BtSP (a, 5 × 10^−5^ M) and BtSP (b, 1 × 10^−4^ M) after addition of 1.0 equiv. of Ca^2+^, Ma^2+^, Zn^2+^, and Ma^2+^, Zn^2+^ metal perchlorates in CH_3_CN at 293 K, respectively; (**c**) Comparison of the absorbance at 495 nm of EG-BtSP and BtSP normalized with the absorbance at 310 nm (the isosbestic point); (**d**) Job’s analysis of the EG-BtSP-Zn^2+^ complex ([EG-BtSP] + [Zn^2+^] = 5 × 10^−5^ M) and BtSP-Zn^2+^ complex ([BtSP] + [Zn^2+^] = 1 × 10^−4^ M) in CH_3_CN; (**e**,**f**) Absorption titration at 493 nm of a solution of EG-BtSP (d, 5 × 10^−5^ M) and BtSP (e, 1 × 10^−4^ M) after increasing the concentration of Zn^2+^ in CH_3_CN; (**g**) Schematic illustration of the equilibria of the EG-BtSP-Zn^2+^ complex. The first equilibrium constant *K*_1_ is much larger than the second *K*_2_. Although the maximum possible coordination number for Zn^2+^ is 6, the coordination occupied by any solvent molecule(s) were omitted for clarity.

Interestingly, the modification of the auxiliary chains appeared to exert slight, but important effects on the sensory property of SP, especially for sensing Ca^2+^. The absorption spectra of EG-BtSP (0.050 mM) and BtSP (0.10 mM) in acetonitrile before and after the addition of 1.0 equiv. of Ca^2+^, Cd^2+^, Co^2+^, Fe^2+^, Mg^2+^, Ni^2+^, Zn^2+^, and Li^+^ metal ions are shown in Figure 1a,b. When certain metal ions (especially Zn^2+^, Mg^2+^, and Ca^2+^) were added, SP molecules switched to the colored forms, presumably due to complex formation with metal ions. The absorption maximum of the complex (~500 nm) was blue-shifted from that of the open MC form (~600 nm), which indicated binding of metal ions to the cleaved phenoxide moiety of SP. To compare the reactivities of SPs based on their auxiliary group, the absorbance at 495 nm, which was normalized to the absorbance at 310 nm to correct for the concentration difference and plotted as a function of different metal ions (Figure 1c). Both SPs showed high selectivity toward Zn^2+^, then to Mg^2+^, among the metal ions tested. Although the general trends for sensory properties of EG-BtSP and BtSP were very similar, EG-BtSP showed a more sensitive and selective response toward Ca^2+^ than BtSP. The only structural difference between EG-BtSP and BtSP was the presence and absence of a glycol moiety, respectively. However, they showed quite different selectivity and sensitivity to Ca^2+^ metal ions. The above results indicate that metal ions could be selectivity regulated by the choice of the auxiliary group to SP molecules and the click reaction to a propargyl-SP **6** should be useful for introducing various functional groups.

Stoichiometries of binding of metal ions to EG-BtSP and BtSP determined by Job plots, a continuous variation method (Figure 1d, Figures S1 and S2). Figure 1d shows that the Job plots of EG-BtSP and BtSP toward Zn^2+^ deviated from normal triangular shapes and appeared as hyperboles. Since the Job plot is based on the assumption that only one complex H_n_G_m_ (H: host, and G: guest molecule) is formed, it may indicate that SP-M^2+^ complexes with several stoichiometries could be present in the solution, or that the binding constants are relatively small [23] Nevertheless, the Job analyses showed that the simple BtSP appeared to have an absorption maximum at a molar fraction of ~0.5, which indicates 1:1 binding to Zn^2+^. The triethylene glycol-functionalized EG-BtSP showed a slight shift toward a higher molar fraction of Zn^2+^ (~0.6), which suggests a predominantly 1:1 binding, but it is also possible that more than one Zn^2+^ may bind to EG-BtSP.

For a better understanding of SP-metal ions binding modes, a UV-VIS spectroscopic titration was performed for EG-BtSP-Ca^2+^, -Mg^2+^, -Zn^2+^ and BtSP-Mg^2+^, and -Zn^2+^ (Figure 1e,f and Figures S3–S7). To the solutions of SP host molecules, various equivalents of metal ions were added, and the absorbance at 495 nm was monitored for each SP-metal ion combination. The absorbance datasets were then subjected to non-linear regression analysis, following a procedure developed by Thordarson’s group [24,25]. Briefly, the datasets were fitted for 1:1 (SP:M^2+^) and 1:2 binding systems and the results were qualified by “*cov*_fit_” values. The *cov*_fit_ values are insensitive to the number of parameters used in the fitting process, and showed a numerical representation of experimental data scatter about the fitted lines. Then the “*cov*_fit_ factor”, which is the *cov*_fit_ value of the 1:1 binding divided by the *cov*_fit_ value of the 1:2 binding, can be used to determine which binding model can provide the best explanation of the experimental data (Table 1). Based on the analyses, all SP-M^2+^ complexes formed at ratios of 1:2 rather than 1:1, as judged by the *cov*_fit_ factors, although the second binding constants (*K*_2_) were much smaller than the first ones (*K*_1_). In addition, triethlyene glycol-functionalized EG-BtSP seems to bind to metal ions slightly stronger when compared to BtSP (which lacks the ethylene glycol moiety): for Zn^2+^, *K*_1_ for EG-BtSP = 85,000 vs. *K*_1_ for BtSP = 72,000, and for Mg^2+^, *K*_1_ for EG-BtSP = 12,000 vs. *K*_1_ for BtSP = 10,000, which corresponds to 0.4~0.5 kJ/mol difference in Δ*G* (Tables S1–S5). We attributed this difference to the participation of the triethylene glycol moiety in the binding of metal ions. In our previous research, we have shown that the phenoxide and methoxy groups in the MC form interact with the metal ion, as well as the triazole part of the open isomer. It is reasonable to assume that lone pair electrons of oxygens in the triethylene glycol moiety would favorably interact with metal ions, resulting in a slightly higher binding constant for EG-BtSP. It should be noted here that the triethylene-glycol auxiliary group of EG-BtSP plays an important role in the Ca^2+^ binding, the binding constant of which is larger than that of Mg^2+^, while the simple BtSP showed little interaction to Ca^2+^.

Based on the analyses above, we proposed the binding scheme of EG-BtSP with Zn^2+^ in Figure 1g. When the first Zn^2+^ ion binds to EG-BtSP, the SP form is transformed to the MC form, and the phenoxy, methoxy, and triazole groups all participate in the binding, as well as the triethylene glycol moiety. When the second Zn^2+^ ion binds to the first complex, we assume that the triazole and triethylene glycol moiety are responsible for binding of the second ion, while the MC form retains the first ion. Since the second binding requires the breaking of triazole- and triethylene glycol-Zn^2+^ interactions, although they are weak, *K*_2_ is much smaller than *K*_1_ (negative cooperativity). We do not know the exact conformation of the triethylene glycol moiety in the binding process, but it is certain that the auxiliary group plays an important role.

**Figure 2:**
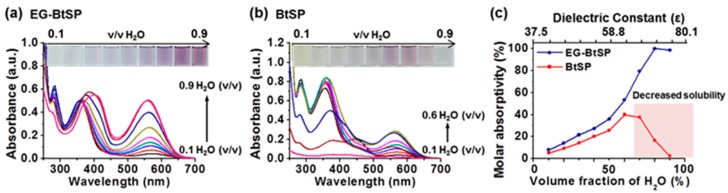
(**a**,**b**) UV-absorption spectra of EG-BtSP (a, c = 5 × 10^−5^ M) and BtSP (b, c = 1 × 10^−4^ M) in different water/CH_3_CN mixtures from 1/1 to 9/1 (*v*/*v*) at 25 °C. Color changes of the solutions were also presented; (**c**) Relative molar absorptivities of EG-BtSP and BtSP at 564 nm were compared according to the volume fraction of water in CH_3_CN.

Modification by an auxiliary group can significantly enhance the utility of SP, especially in an aqueous environment. It was shown that EG-BtSP could be dissolved in the solvent at a water/acetonitrile ratio of up to 9:1. As shown in Figure 2a, EG-BtSP was transformed to the MC form with increasing amounts of water in the co-solvent system. We attributed this transformation to the higher dielectric environment of water, which can enhance the stabilization of strong dipoles of the open MC form (zwitterion). BtSP could also be isomerized to the MC form by increasing the amount of water. However, BtSP was precipitated at water/acetonitrile ratios of 60/40 (60%), indicating poor solubility in water (Figure 2b). As shown in Figure 2c, the dielectric constant of the co-solvent system increased the transformation of both SPs to their MC form. However, the ethylene-glycol auxiliary chain clearly helped the transformation and the stability of EG-BtSP in an aqueous environment.

**Figure 3:**
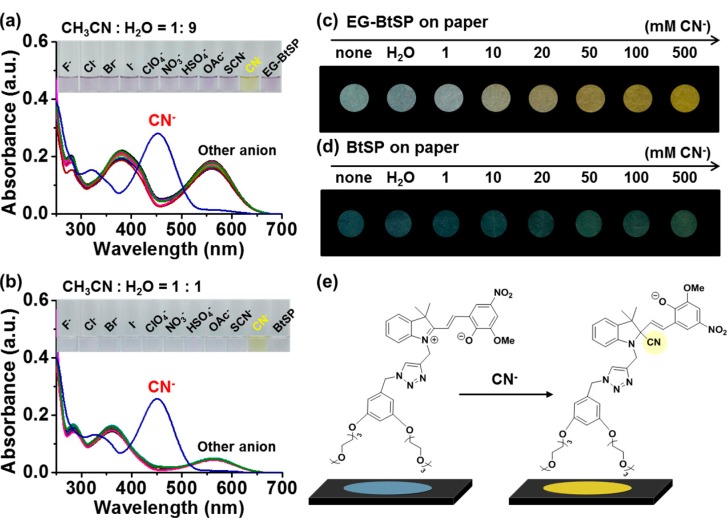
(**a**,**b**) UV-VIS absorption spectra of EG-BtSP (a, 2 × 10^−5^ M) and BtSP (b, 2 × 10^−5^ M) measured with 50 equiv. of respective anion (as a *n*-Bu_4_N^+^ salt) in a water/CH_3_CN mixture (9:1 or 1:1 *v*/*v*) at 25 °C. The spectra were obtained 30 min after addition of the anion to the SP solution. Photographs of the solutions were also presented: from left F^−^, Cl^−^, Br^−^, I^−^, ClO_4_^−^, NO_3_^−^, HSO_4_^−^, OAc^−^, CN^−^, without any anion; (**c**,**d**) Colorimetric changes of the papers with SP probes, EG-BtSP (**c**) and BtSP (**d**); upon the application of cyanide in H_2_O. From left to right: probe only, H_2_O, 1 mM, 10 mM, 20 mM, 50 mM, 100 mM, and 500 mM of cyanide; (**e**) Schematic illustration of paper-based colorimetric detection of cyanide with EG-BtSP.

The enhanced stability of EG-BtSP in an aqueous environment by auxiliary-chain modification enabled the fabrication of paper-based colorimetric sensors for a cyanide ion. As reported in the literature, the zwitterionic MC form has an electrophilic site that nucleophiles (e.g., cyanide ion) can attack [26]. However, most SP sensors for the cyanide ion have been tested in organic solvents and only a few examples have been tested in water/acetonitrile co-solvent, as previously reported elsewhere [26,27,28] Moreover, no paper-based sensor with SP molecules has been developed yet. We investigated the sensory responses of EG-BtSP and BtSP toward various anions in solution. For screening, the absorption spectra of EG-BtSP (0.020 mM) in a 9:1 water/acetonitrile co-solvent system were measured after addition of F^−^, Cl^−^, Br^−^, I^−^, ClO_4_^−^, NO_3_^−^, HSO_4_^−^, OAc^−^, and CN^−^ (50 equiv.). As shown in Figure 3a, only the addition of CN^−^ induced the blue-shift of λ_max_ in the absorption spectrum of EG-BtSP (from 560 nm to 453 nm); the violet color of the initial EG-BtSP solution turned to a yellowish hue. Due to the poor solubility of BtSP in water, we tested its sensory response at a concentration of 0.02 mM in a 1:1 ratio of water:acetonitrile. As shown in Figure 3b, BtSP also showed a selective response to the cyanide ion. It should be noted here that, unlike BtSP, EG-BtSP showed its cyanide-selective response in a mostly aqueous environment. The effect of auxiliary modification was mostly reflected in the fabrication of SP-utilized paper-based sensors. Pre-cut filter papers were dip-coated with a solution of EG-BtSP or BtSP (100 mM) and then exposed to different concentrations of aqueous cyanide solutions (from 1 mM to 500 mM). As shown in Figure 3c,d, the EG-BtSP-incorporated paper sensors showed apparent color changes (bluish-violet to yellow) after applying aqueous cyanide ions. However, the BtSP-incorporated paper sensors showed very little sensory responses toward aqueous cyanide ions, even at a concentration of 500 mM. In solution, BtSP also operated as a cyanide sensor (Figure S8) similar to EG-BtSP, but on the paper, its sensory response was minimal. When we performed the CN^−^ sensing test for BtSP in acetonitrile-water 1:1 mixture, a distinct color change was observed over 50 mM of CN^−^ (Figure S9). This is because the insolubility issue of BtSP was somewhat resolved in the acetonitrile-water mixture. However, it should be noted here that the purpose of our study was to detect CN^−^ in a purely aqueous environment. Due to its limited solubility in water, BtSP did not respond to CN^−^ in water only, which highlights the importance of the auxiliary group. The EG-BTSP-incorporated paper sensor showed a dramatic color change toward cyanide ions with the help of the hydrophilic auxiliary chain of triethylene glycols. Interesting CN^−^-selective paper strip-based sensors with probe molecules based on benzothiazolyl-malononitrile by Hong group [29] and densyl-triazole-glucopyranosyl conjugates by Rao group [30] were separately reported, but they are based on new fluorescence emission or its enhancement, respectively, upon addition of CN^−^. However, our paper-based SP sensors were colorimetric, which is easy to apply and rapidly detects in a cost-effective manner. Furthermore, our paper-based SP sensor could work in purely aqueous environment. Chow, Tang, and coworkers reported effective colorimetric paper-based sensor with an ethenyl-allylpyridinum derivative, but they utilized an acetonitrile-water (95:5, *v*/*v*) mixture [31].

## 4. Conclusions

The triethylene glycol-functionalized EG-BtSP and the simple BtSP were synthesized from propargyl-SP **6** via a click reaction, and both SPs were investigated their new sensory properties following the regulation of the SP auxiliary group. Both SPs showed similar sensitivities to Mg^2+^ and Zn^2+^, but EG-BtSP demonstrated a better sensitivity to Ca^2+^ than BtSP. Higher dielectric constants of the solvent mixtures (water/acetonitrile) were associated with the presence of more zwitterionic forms of both SPs. However, BtSP precipitated at ratios of water/acetonitrile of over 60/40, while EG-BtSP remained stable at ratios of water/acetonitrile of up to 90/10, due to improved hydrophilicity conferred by the presence of a triethylene-glycol auxiliary group. Additionally, in solution, both EG-BtSP and BtSP showed similar selective sensory responses to cyanide. However, when fabricated on paper, only EG-BtSP showed an apparent color change. We showed that the sensory properties of SP molecules could be easily tuned by auxiliary groups, and click chemistry enabled the facile introduction of appropriate auxiliary groups from propargyl-functionalized SPs.

## Figures and Tables

**Table 1:** Plausible binding model, *K*_1_, *K*_2_, and interaction parameter (α), and relative quality of fit for the complexation of EG-BtSP and BtSP toward metal ions obtained from UV-VIS spectroscopy at 298 K in acetonitrile ^a^.

Spiropyran-Metal		Binding Model	*cov*_fit_ Factor ^b^	*K*_1_ (M^−1^)	*K*_2_ (M^−1^)	α ^c^
EG-BtSP	Ca^2+^	1:2	22.8	4.73 × 10^4^	1803	0.161
	Mg^2+^	1:2	11.7	1.20 × 10^4^	353	0.118
	Zn^2+^	1:2	64.7	8.52 × 10^4^	868	0.041
BtSP	Mg^2+^	1:2	34.8	9.95 × 10^3^	172	0.074
	Zn^2+^	1:2	48.0	7.20 × 10^4^	636	0.034

^a^ The data here are the rounded averages of the triplicate measurements. The fittings with the experimental data are shown in detail in Supporting Information (Tables S1–S5). ^b^ The relative quality of the fit could be expressed in terms of *cov*_fit_ factor = *cov*_fit_ for the 1:1 model divided by the *cov*_fit_ for the 1:2 model, where *cov*_fit_ is the (co)variance of the residuals divided by the (co)variances of the raw data. ^c^ The interaction parameter α = 4*K*_2_/*K*_1_ with α > 1 indicating positive cooperativity, α < 1 negative cooperativity, and α = 1 no cooperativity [24,25].

## Supplementary Materials

The Supplementary Materials are available online at http://www.mdpi.com/1424-8220/17/8/1816/s1.
