# Supplementary material for: Tuning Sensory Properties of Triazole-Conjugated Spiropyrans: Metal-Ion Selectivity and Paper-Based Colorimetric Detection of Cyanide
Source: Sensors (Basel). 2017 Aug 7;17(8):1816. doi: 10.3390/s17081816 (PMC5579521; doi:10.3390/s17081816)
Supplement: Supplementary file 1 [file sensors-17-01816-s001.pdf]

Supplementary Material for

Tuning Sensory Properties of Triazole-Conjugated Spiropyrans: Metal-Ion Selectivity and Paper-Based Colorimetric Detection of Cyanide

Juhyen Lee,[a] Eun Jung Choi,[a] Inwon Kim,[a,†] Minhe Lee,[a] Chinnadurai Satheeshkumar,[b] and Changsik Song,[a]*

[a]Department of Chemistry, Sungkyunkwan University, Suwon, Gyeonggi, 16419, Republic of Korea. [b]Graduate School of Nanoscience and Technology, Korea Advanced Institute of Sciencce and Technology (KAIST), Daejeon, 34141, Republic of Korea

Email: songcs@skku.edu

[†]Present address: Department of Chemistry, KAIST and Center for Catalytic Hydrocarbon Functionalizations, Institute for Basic Science (IBS), Daejeon, 34141, Korea

Contents:

Figure S1. Job's analyses of (a) **EG-BtSP-Ca^{2+}**, (b) **EG-BtSP-Mg^{2+}**, and (c) **EG-BtSP-Zn^{2+}** complexes in CH_3CN. p 2

Figure S2. Job's analyses of (a) **BtSP-Mg^{2+}** and (b) **BtSP-Zn^{2+}** complexes in CH_3CN. p 2

Tables S1–S3 and **Figures S3–S5.** The UV-vis spectroscopic titrations of **EG-BtSP** with Ca^{2+}, Mg^{2+}, and Zn^{2+}, followed by nonlinear regression analysis for binding models (a) **1:1** and (b) **1:2**. p 3-5

Tables S4–S5 and **Figures S6–S7.** The UV-vis spectroscopic titrations of **BtSP** with Mg^{2+} and Zn^{2+}, followed by nonlinear regression analysis for binding models (a) **1:1**, (b) **1:2**. p 6-7

Figure S8. The UV-vis absorption spectra of solutions of **EG-BtSP** and **BtSP** after the addition of different amounts of cyanide (up to 10 equiv). p 8

Figure S9. The colorimetric changes of the papers with **BtSP** probes upon the Application of cyanide in acetonitrile-water (1:1 v/v) mixture solution. p 8

Figures S10–S21. ^{1}H and ^{13}C NMR spectra of compounds **1**, **2**, **3**, **6**, **EG-BtSP** and **BtSP**. p 9-14

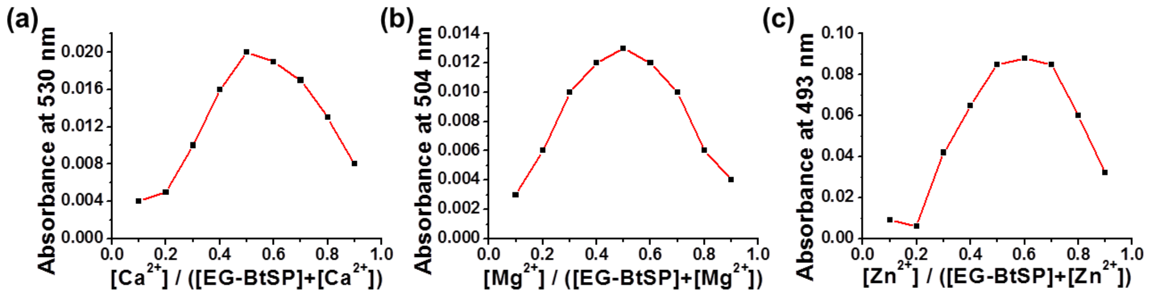

Figure S1. Job's analyses of (a) **EG-BtSP-Ca^{2+}** complex, (b) **EG-BtSP-Mg^{2+}** complex, and (c) **EG-BtSP-Zn^{2+}** complex. [**EG-BtSP**] + [M^{2+}] = 5 × 10^{-5} M and all solutions were in CH_3CN.

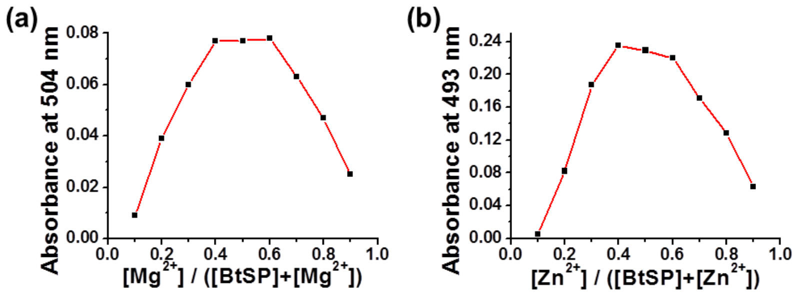

Figure S2. Job's analyses of (a) **BtSP-Mg^{2+}** complex and (b) **BtSP-Zn^{2+}** complex. [**BtSP**] + [M^{2+}] = 1 × 10^{-4} M and both solutions were in CH_3CN.

Table S1. Association constants of **EG-BtSP** (5×10^{-5} M, CH_3CN) towards the calcium cation obtained from UV-vis spectroscopic titration and nonlinear regression analysis.

binding model	experiment #	cov_{fit} (10^{-3})	cov_{fit} factor	K_1 (M^{-1})	K_2 (M^{-1})	β_{12} (M^{-2})	ΔG_1 (kJ/mol)	ΔG_2 (kJ/mol)	α ($4K_2/K_1$)
	1	12.3	1	0.96×10^4	-	-	-22.7	-	-
	2	15.3	1	1.15×10^4	-	-	-23.2	-	-
	3	14.2	1	1.30×10^4	-	-	-23.5	-	-
1:1	**Average**	**13.9**	**1**	**1.14×10^4**	**-**	**-**	**-23.1**	**-**	**-**
	Std. Dev.	1.52	-	0.17×10^4	-	-	0.38	-	-
	95% C.I.	3.43	-	0.39×10^4 (34%)	-	-	0.86	-	-
	1	0.70	17.6	2.91×10^4	1480	4.31×10^7	-25.5	-18.1	0.203
	2	0.50	30.6	4.33×10^4	1549	6.70×10^7	-26.4	-18.2	0.143
	3	0.64	22.2	6.95×10^4	2380	16.5×10^7	-27.6	-19.3	0.137
1:2	**Average**	**0.61**	**22.8**	**4.73×10^4**	**1803**	**9.18×10^7**	**-26.5**	**-18.5**	**0.161**
	Std. Dev.	0.10	-	2.05×10^4	501	6.48×10^7	1.08	0.65	0.040
	95% C.I.	0.23	-	4.64×10^4 (98%)	1133 (63%)	14.7×10^7	2.44	1.47	0.083

[a]cov_{fit} factor = cov_{fit} for the **1:1** model divided by the cov_{fit} for the **1:2** binding model. The analyses (Tables S1–S5) were followed by Thordarson *et al. J. Am. Chem. Soc.* **2014,** *136,* 7505-7516. The details were described therein.

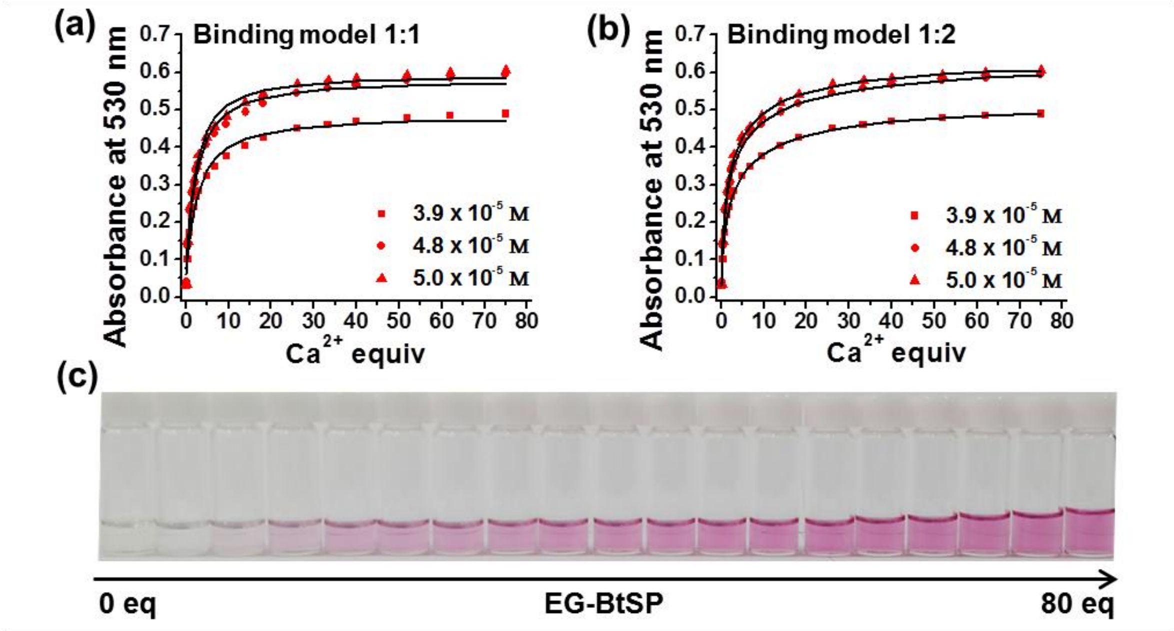

Figure S3. (a-b) UV-vis spectroscopic titration of **EG-BtSP** (5×10^{-5} M, CH_3CN) with $Ca(ClO_4)_2$ and their fitting results with a non-linear regression method of the binding model (a) **1:1** and (b) **1:2**. (c) Color changes of **EG-BtSP** solutions during the titration.

Conclusion: The binding model **1:2** is always much better than the binding model **1:1**.

Table S2. Association constants of **EG-BtSP** (5×10^{-5} M, CH_3CN) towards the magnesium cation obtained from UV-vis spectroscopic titration and nonlinear regression analysis.

binding model	experiment #	cov_{fit} (10^{-3})	cov_{fit} factor	K_1 (M^{-1})	K_2 (M^{-1})	β_{12} (M^{-2})	ΔG_1 (kJ/mol)	ΔG_2 (kJ/mol)	α ($4K_2/K_1$)
	1	16.5	1	4.52×10^3	-	-	-20.9	-	-
	2	10.7	1	5.24×10^3	-	-	-21.2	-	-
	3	16.4	1	6.19×10^3	-	-	-21.6	-	-
1:1	**Average**	**14.5**	**1**	$\mathbf{5.32 \times 10^3}$	**-**	**-**	**-21.2**	**-**	**-**
	Std. Dev.	3.32	-	0.84×10^3	-	-	0.39	-	-
	95% C.I.	7.51	-	1.89×10^3 (36%)	-	-	0.88	-	-
	1	0.68	24.3	1.01×10^4	230	2.32×10^6	-22.8	-13.5	0.091
	2	1.12	9.55	1.12×10^4	428	4.80×10^6	-23.1	-15.0	0.153
	3	1.91	8.59	1.47×10^4	400	5.86×10^6	-23.8	-14.8	0.109
1:2	**Average**	**1.24**	**11.7**	$\mathbf{1.20 \times 10^4}$	**353**	$\mathbf{4.33 \times 10^6}$	**-23.2**	**-14.4**	**0.118**
	Std. Dev.	0.62	-	0.24×10^4	107	1.82×10^6	0.48	0.84	0.030
	95% C.I.	1.41	-	0.54×10^4 (45%)	243 (69%)	4.11×10^6	1.08	1.91	0.072

[a]cov_{fit} factor = cov_{fit} for the **1:1** model divided by the cov_{fit} for the **1:2** binding model.

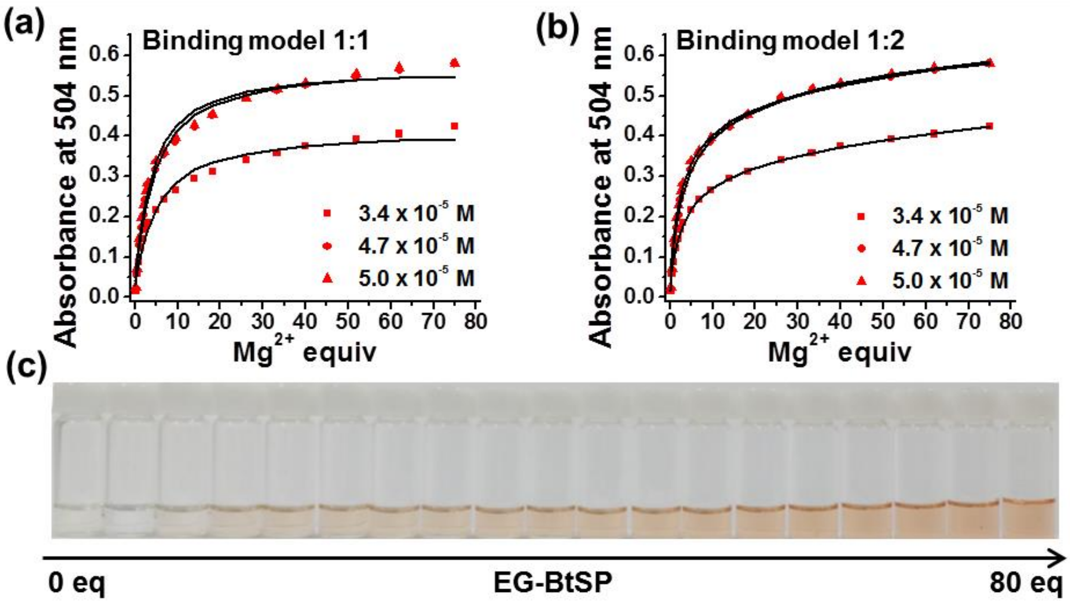

Figure S4. (a-b) UV-vis spectroscopy titration of **EG-BtSP** (5×10^{-5} M, CH_3CN) with $Mg(ClO_4)_2$ and their fitting result with a non-linear regression method of the binding model (a) **1:1** and (b) **1:2**. (c) Color changes of **EG-BtSP** solutions during the titration.

Conclusion: The binding model **1:2** is always much better than the binding model **1:1**.

Table S3. Association constants of **EG-BtSP** (5×10^{-5} M, CH_3CN) towards the zinc cation obtained from UV-vis spectroscopic titration and nonlinear regression analysis.

binding model	experiment #	cov_{fit} (10^{-3})	cov_{fit} factor	K_1 (M^{-1})	K_2 (M^{-1})	β_{12} (M^{-2})	ΔG_1 (kJ/mol)	ΔG_2 (kJ/mol)	α ($4K_2/K_1$)
1:1	1	49.2	1	8.23×10^3	-	-	-22.3	-	-
	2	47.6	1	9.24×10^3	-	-	-22.6	-	-
	3	43.1	1	7.94×10^3	-	-	-22.2	-	-
	**Average**	**46.6**	**1**	$\mathbf{8.47 \times 10^3}$	**-**	**-**	**-22.4**	**-**	**-**
	Std. Dev.	3.16	-	0.68×10^3	-	-	0.20	-	-
	95% C.I.	7.16	-	1.54×10^3 (18%)	-	-	0.44	-	-
1:2	1	0.84	58.6	10.0×10^4	938	9.40×10^7	-28.5	-17.0	0.037
	2	1.21	39.3	8.87×10^4	888	7.87×10^7	-28.2	-16.8	0.040
	3	0.12	359	6.69×10^4	777	5.20×10^7	-27.5	-16.5	0.046
	**Average**	**0.72**	**64.7**	$\mathbf{8.52 \times 10^4}$	**868**	$\mathbf{7.49 \times 10^7}$	**-28.1**	**-16.8**	**0.041**
	Std. Dev.	0.55	-	1.69×10^4	82.4	2.13×10^7	0.51	0.24	0.005
	95% C.I.	1.25	-	3.83×10^4 (45%)	186 (21%)	4.82×10^7	1.16	0.54	0.011

[a] cov_{fit} factor = cov_{fit} for the **1:1** model divided by the cov_{fit} for the **1:2** binding model.

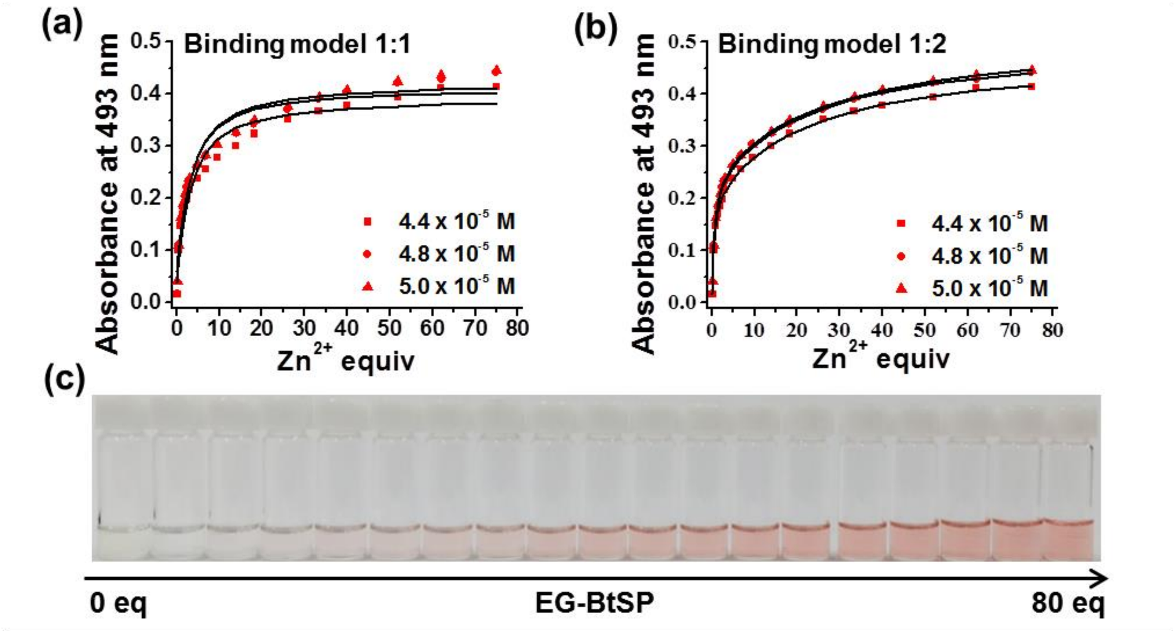

Figure S5. (a-b) UV-vis spectroscopy titration of **EG-BtSP** (5×10^{-5} M, CH_3CN) with $Zn(ClO_4)_2$ and their fitting result with a non-linear regression method of the binding model (a) 1:1 and (b) 1:2. (c) Color changes of **EG-BtSP** solutions during the titration.

Conclusion: The binding model **1:2** is always much better than the binding model **1:1**.

Table S4. Association constants of **BtSP** (1×10^{-4} M, CH_3CN) towards the magnesium cation obtained from UV-vis spectroscopic titration and nonlinear regression analysis.

binding model	experiment #	cov_{fit} (10^{-3})	cov_{fit} factor	K_1 (M^{-1})	K_2 (M^{-1})	β_{12} (M^{-2})	ΔG_1 (kJ/mol)	ΔG_2 (kJ/mol)	α ($4K_2/K_1$)
1:1	1	16.6	1	3.96×10^3	-	-	-20.5	-	-
	2	11.4	1	3.47×10^3	-	-	-20.2	-	-
	3	32.7	1	4.07×10^3	-	-	-20.6	-	-
	**Average**	**20.2**	**1**	**3.83×10^3**	**-**	**-**	**-20.4**	**-**	**-**
	Std. Dev.	11.1	-	0.32×10^3	-	-	0.21	-	-
	95% C.I.	25.1	-	0.73×10^3 (19%)	-	-	0.48	-	-
1:2	1	0.91	18.2	9.61×10^3	202	1.94×10^6	-22.7	-13.2	0.084
	2	0.65	17.5	7.03×10^3	165	1.16×10^6	-21.9	-12.7	0.094
	3	0.17	192	13.2×10^3	148	1.96×10^6	-23.5	-12.4	0.045
	**Average**	**0.58**	**34.8**	**9.95×10^3**	**172**	**1.69×10^6**	**-22.7**	**-12.7**	**0.074**
	Std. Dev.	0.38	-	3.11×10^3	27.6	0.46×10^6	0.78	0.39	0.026
	95% C.I.	0.85	-	7.03×10^3 (71%)	62.5 (36%)	1.03×10^6	1.77	0.89	0.059

[a]cov_{fit} factor = cov_{fit} for the **1:1** model divided by the cov_{fit} for the **1:2** binding model.

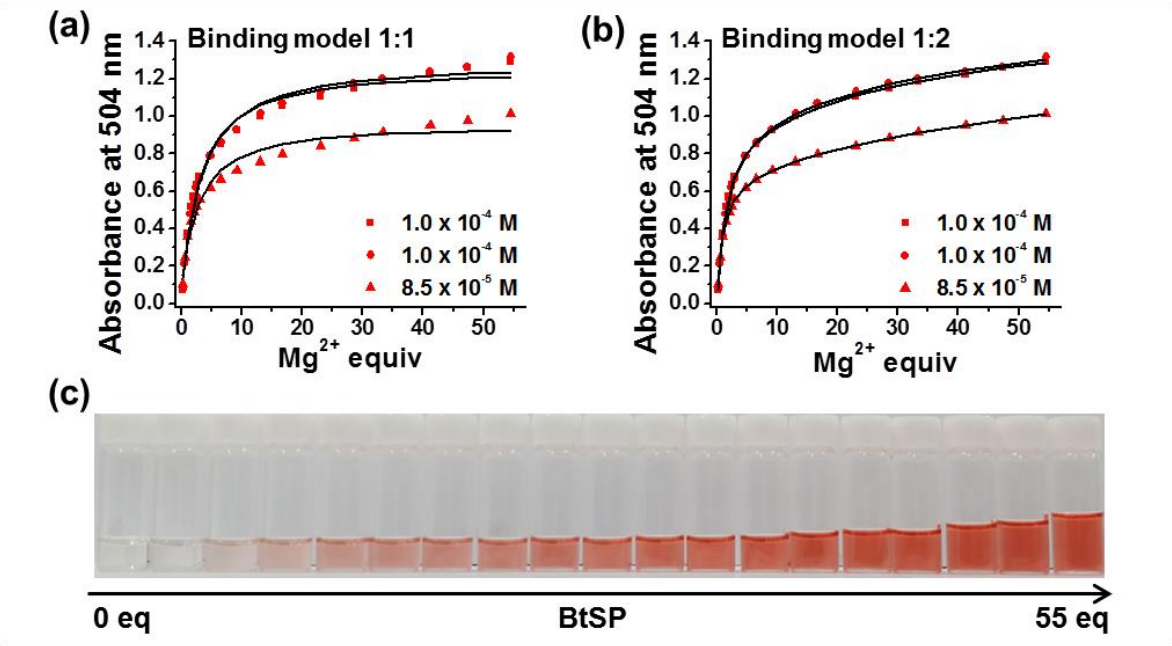

Figure S6. (a-b) UV-vis spectroscopy titration of **BtSP** (1×10^{-4} M, CH_3CN) with $Mg(ClO_4)_2$ and their fitting result with a non-linear regression method of the binding model (a) **1:1** and (b) **1:2**. (c) Color changes of **BtSP** solutions during the titration.

Conclusion: The binding model **1:2** is always much better than the binding model **1:1**.

Table S5. Association constants of **BtSP** (1×10^{-4} M, CH_3CN) towards the zinc cation obtained from UV-vis spectroscopic titration and nonlinear regression analysis.

binding model	experiment #	cov_{fit} (10^{-3})	cov_{fit} factor	K_1 (M^{-1})	K_2 (M^{-1})	β_{12} (M^{-2})	ΔG_1 (kJ/mol)	ΔG_2 (kJ/mol)	α ($4K_2/K_1$)
	1	31.5	1	5.62×10^3	-	-	-21.4	-	-
	2	38.7	1	6.23×10^3	-	-	-21.6	-	-
	3	58.0	1	3.20×10^3	-	-	-20.0	-	-
1:1	**Average**	**42.7**	**1**	$\mathbf{5.02 \times 10^3}$	**-**	**-**	**-21.0**	**-**	**-**
	Std. Dev.	13.7	-	1.61×10^3	-	-	0.89	-	-
	95% C.I.	31.0	-	3.63×10^3 (72%)	-	-	2.01	-	-
	1	1.27	24.8	9.13×10^4	887	8.09×10^7	-28.3	-16.8	0.039
	2	0.96	40.3	7.22×10^4	677	4.89×10^7	-27.7	-16.1	0.037
	3	0.43	135	5.27×10^4	343	1.81×10^7	-26.9	-14.5	0.026
1:2	**Average**	**0.89**	**48.0**	$\mathbf{7.20 \times 10^4}$	**636**	$\mathbf{4.93 \times 10^7}$	**-27.7**	**-15.8**	**0.034**
	Std. Dev.	0.42	-	1.93×10^4	274	3.14×10^7	0.68	1.21	0.007
	95% C.I.	0.96	-	4.37×10^4 (61%)	621 (98%)	7.12×10^7	1.55	2.75	0.016

[a]cov_{fit} factor = cov_{fit} for the **1:1** model divided by the cov_{fit} for the **1:2** binding model.

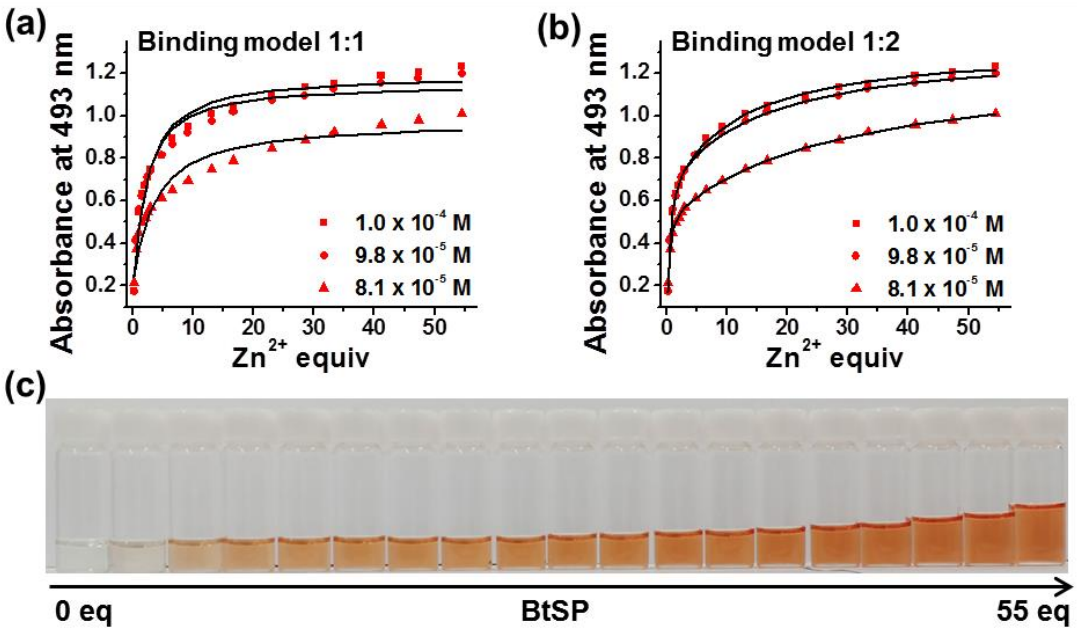

Figure S7. (a-b) UV-vis spectroscopy titration of **BtSP** (1×10^{-4} M, CH_3CN) with $Zn(ClO_4)_2$ and their fitting result with a non-linear regression method of the binding model (a) **1:1** and (b) **1:2**. (c) Color changes of **BtSP** solutions during the titration.

Conclusion: The binding model **1:2** is always much better than the binding model **1:1**.

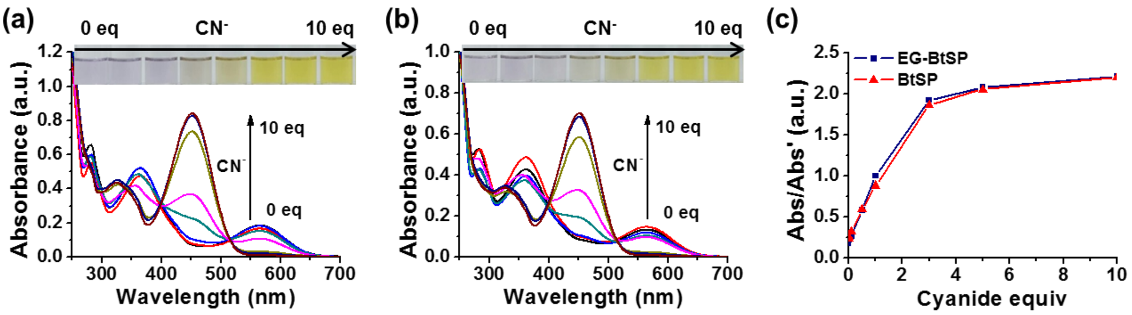

Figure S8. (a, b) The UV-vis absorption spectra of a solution of (a) **EG-BtSP** or (b) **BtSP** (5×10^{-5} M) measured with different concentrations of cyanide (up to 10 equiv) as a potassium salt in water/acetonitrile mixture (1/1 v/v). (c) The plotting of cyanide reactivity (the ratio of absorbance at 450 nm over that at 400 nm) of **EG-BtSP** and **BtSP** demonstrates their reactivities were similar in solutions.

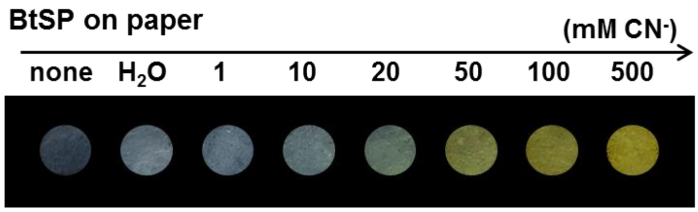

Figure S9. Colorimetric changes of the papers with SP probes, **BtSP** upon the application of cyanide in CH_3CN:H_2O mixture. From left to right: probe only, H_2O, 1 mM, 10 mM, 20 mM, 50 mM, 100 mM, and 500 mM of cyanide.

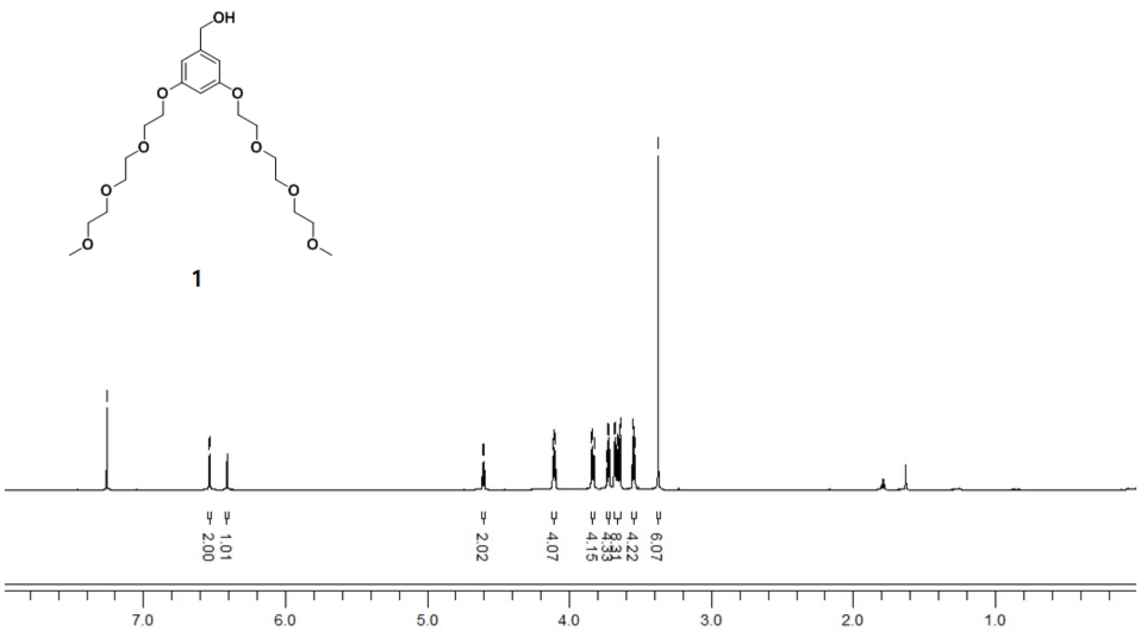

Figure S10. ^{1}H NMR spectrum of **1** (500 MHz, $CDCl_3$).

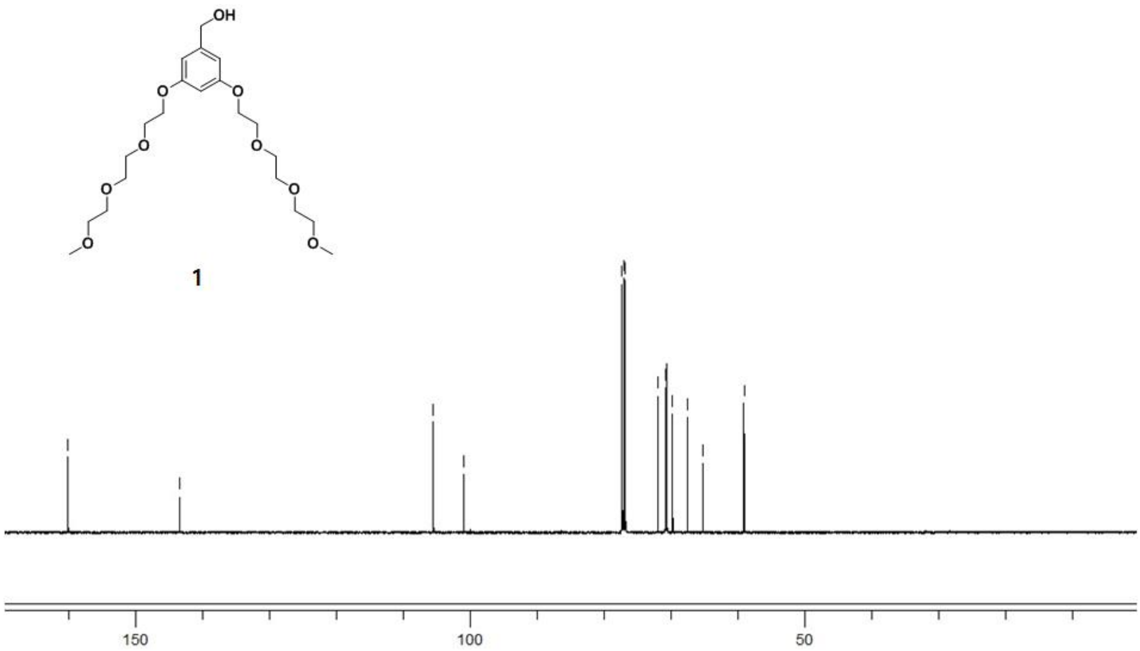

Figure S11. ^{13}C NMR spectrum of **1** (125 MHz, $CDCl_3$).

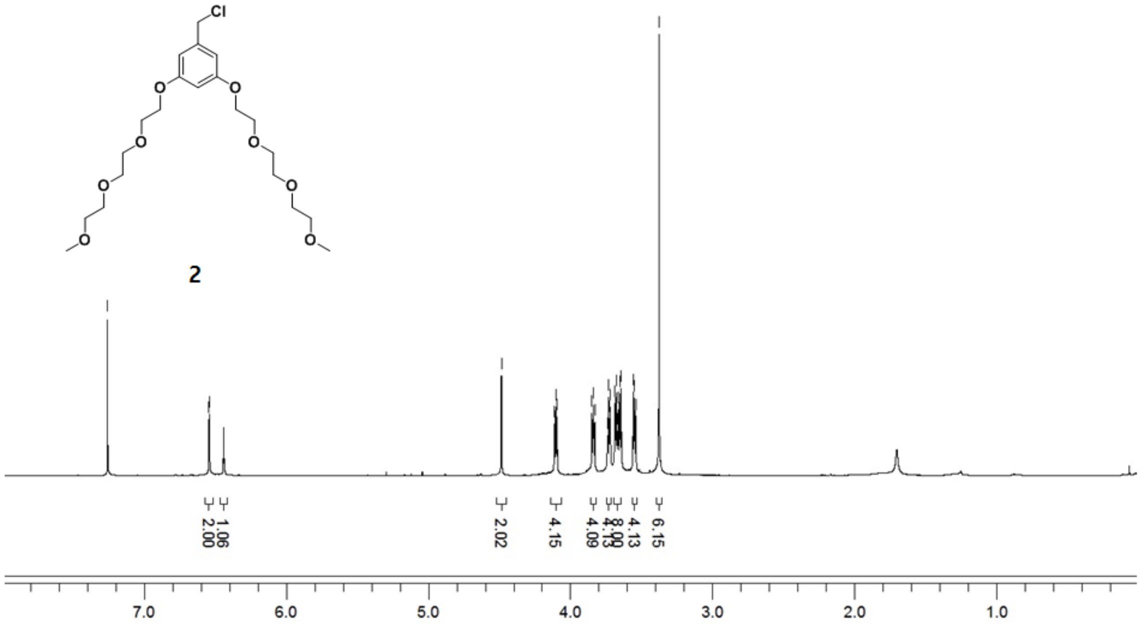

Figure S12. ^{1}H NMR spectrum of **2** (500 MHz, $CDCl_3$).

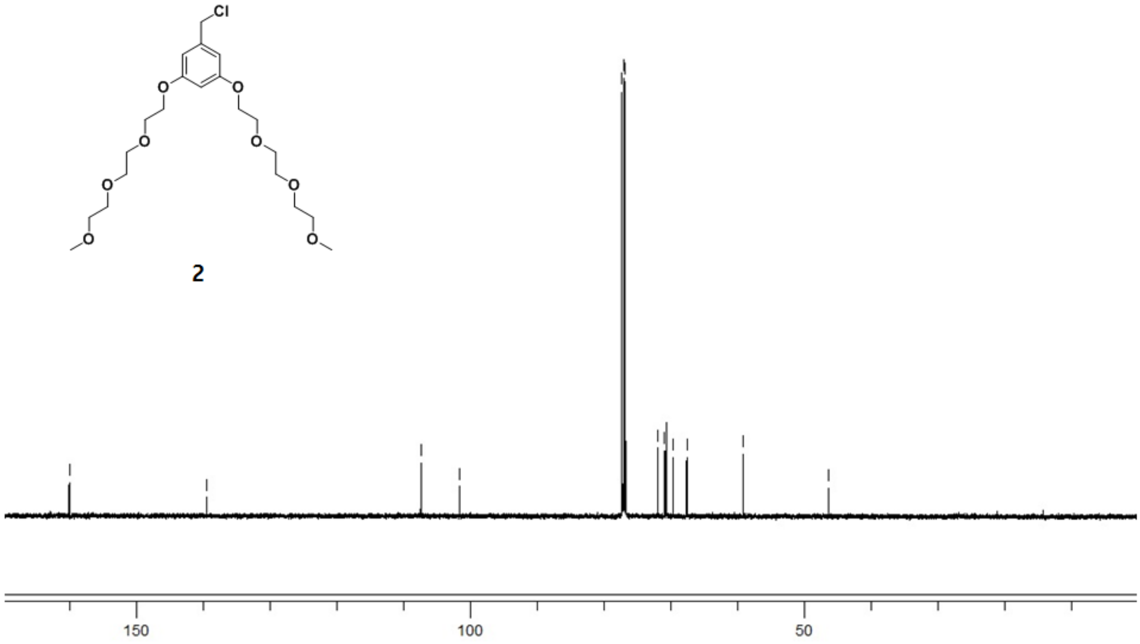

Figure S13. ^{13}C NMR spectrum of **2** (125 MHz, $CDCl_3$).

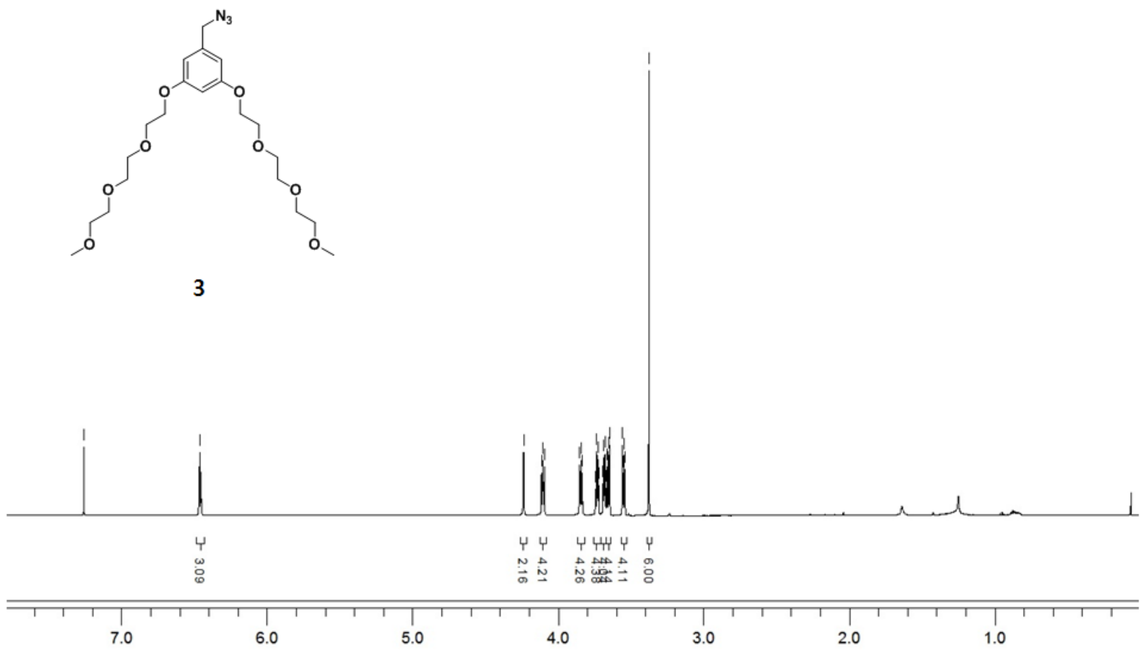

Figure S14. ^{1}H NMR spectrum of **3** (500 MHz, $CDCl_3$).

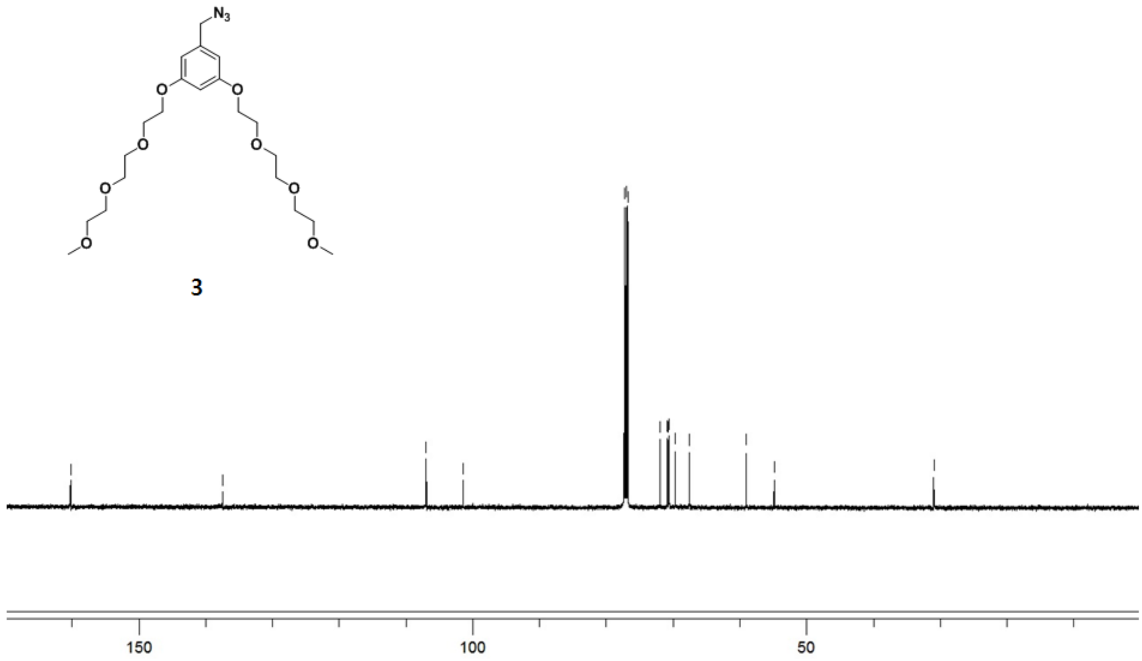

Figure S15. ^{13}C NMR spectrum of **3** (125 MHz, $CDCl_3$).

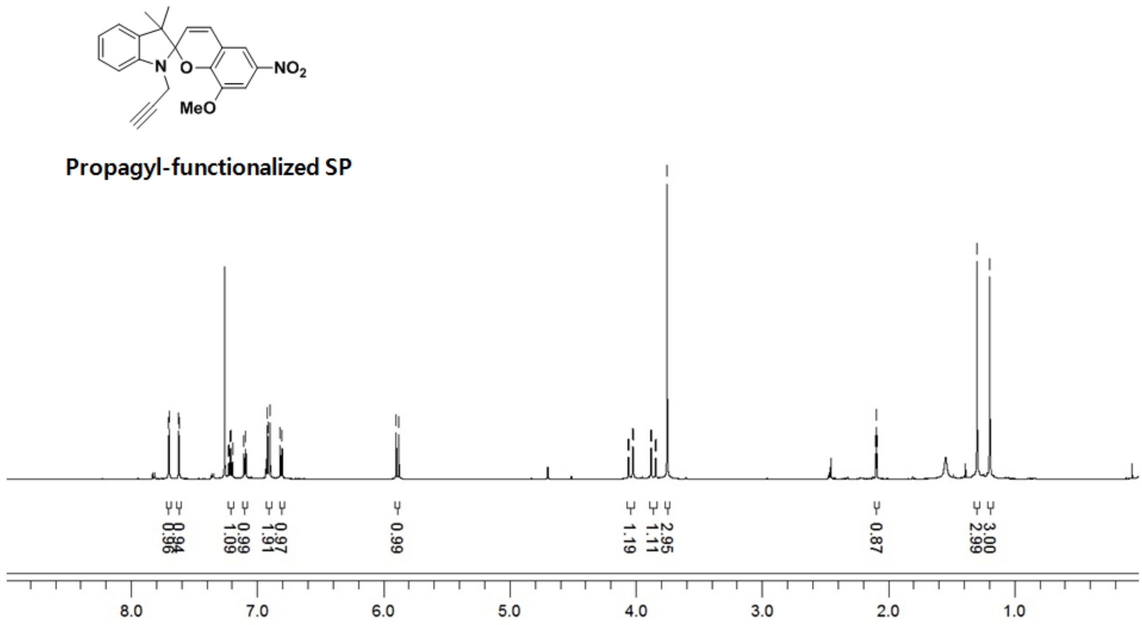

Figure S16. ^{1}H NMR spectrum of **6** (500 MHz, $CDCl_3$).

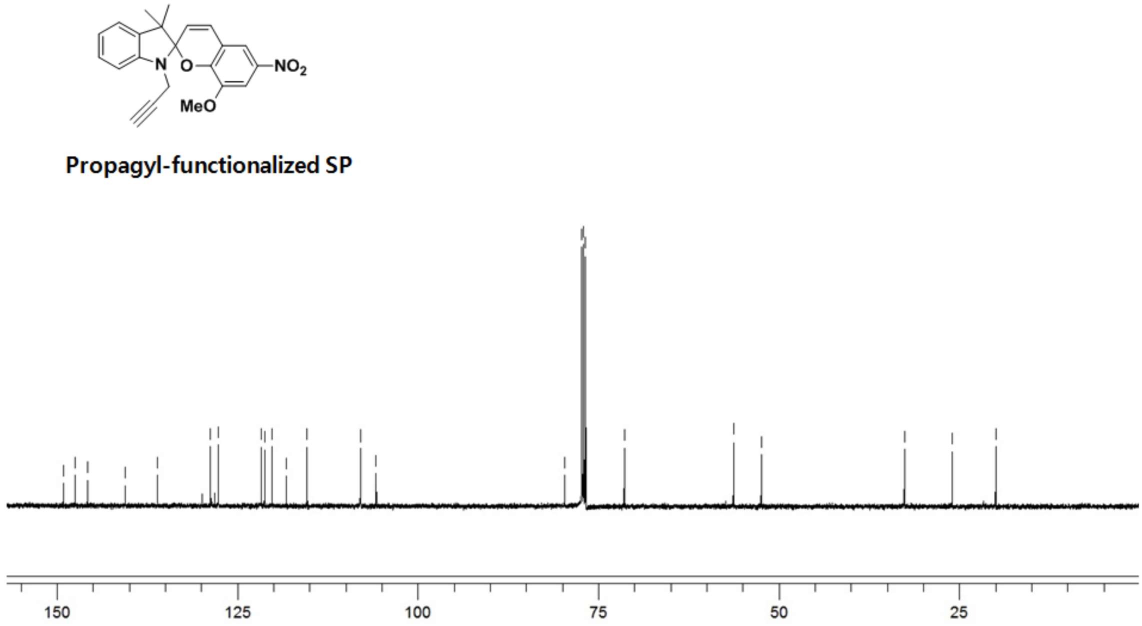

Figure S17. ^{13}C NMR spectrum of **6** (125 MHz, $CDCl_3$).

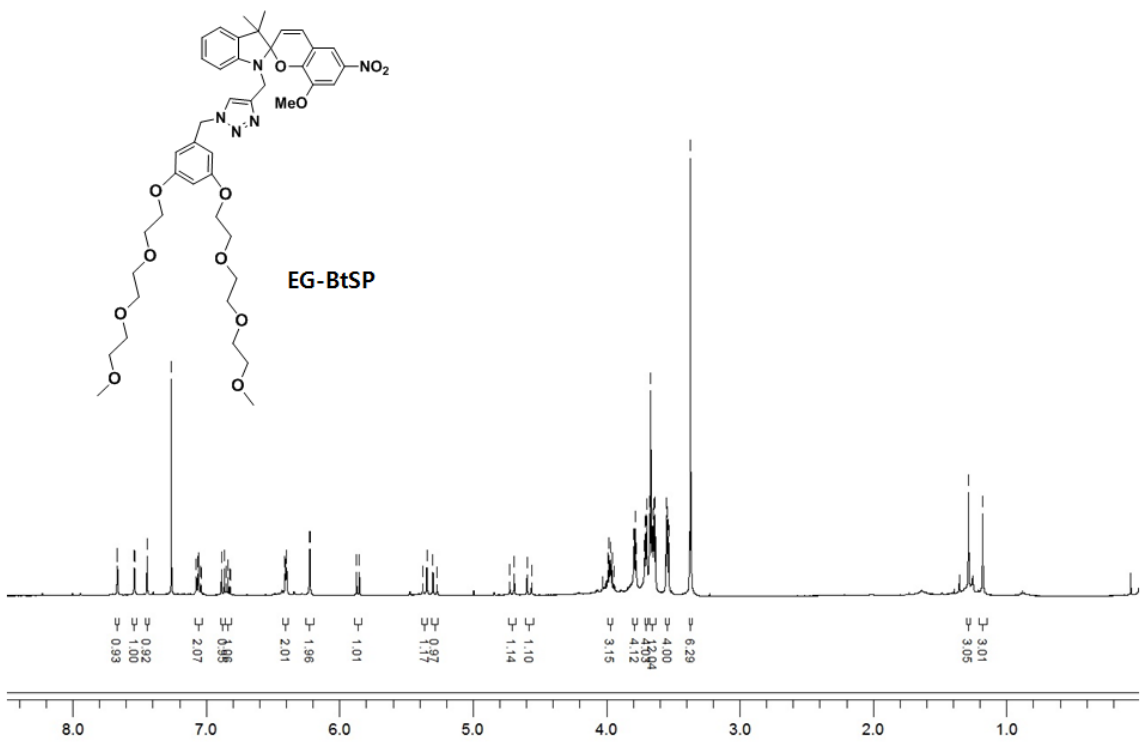

Figure S18. ^1H NMR spectrum of **EG-BtSP** (500 MHz, $CDCl_3$).

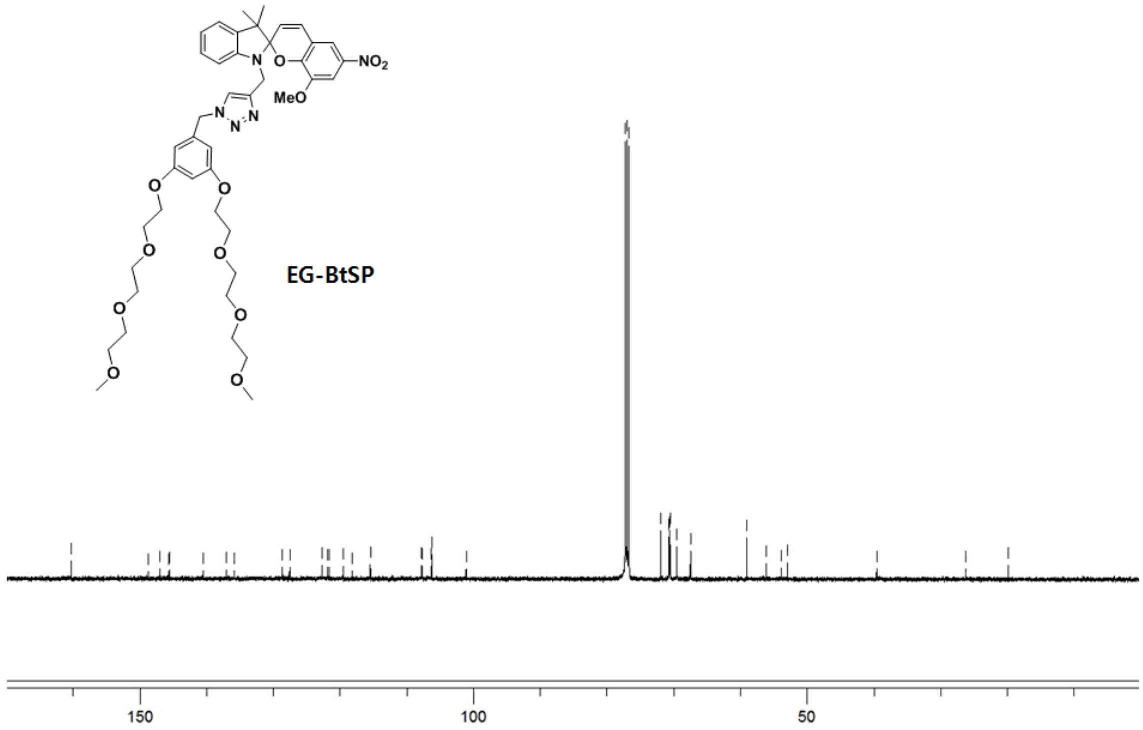

Figure S19. ^{13}C NMR spectrum of **EG-BtSP** (125 MHz, $CDCl_3$).

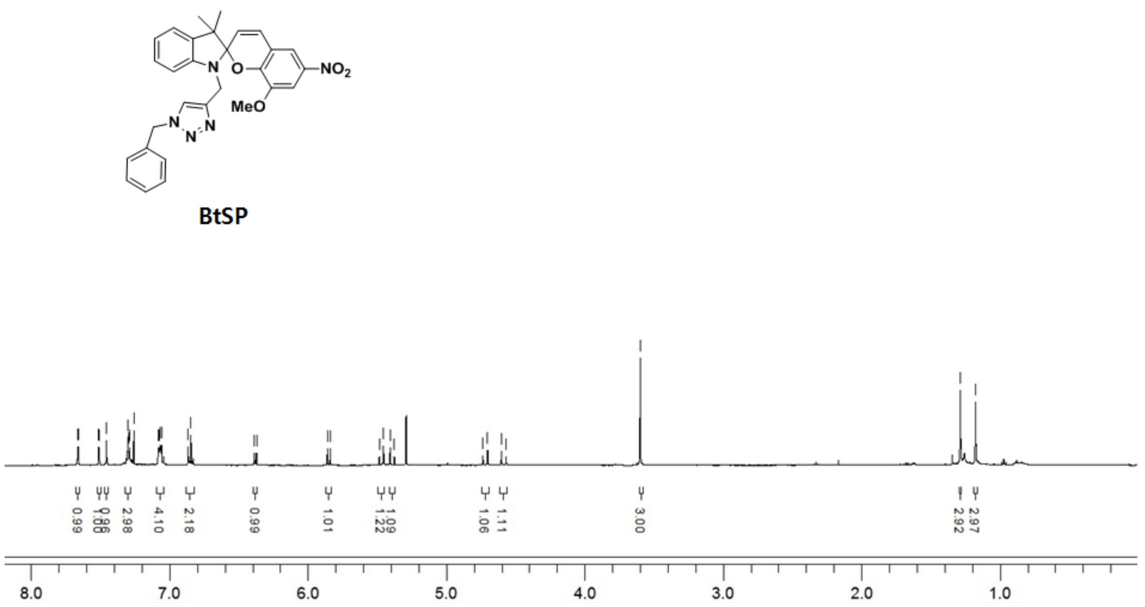

Figure S20. ^{1}H NMR spectrum of **BtSP** (500 MHz, $CDCl_3$).

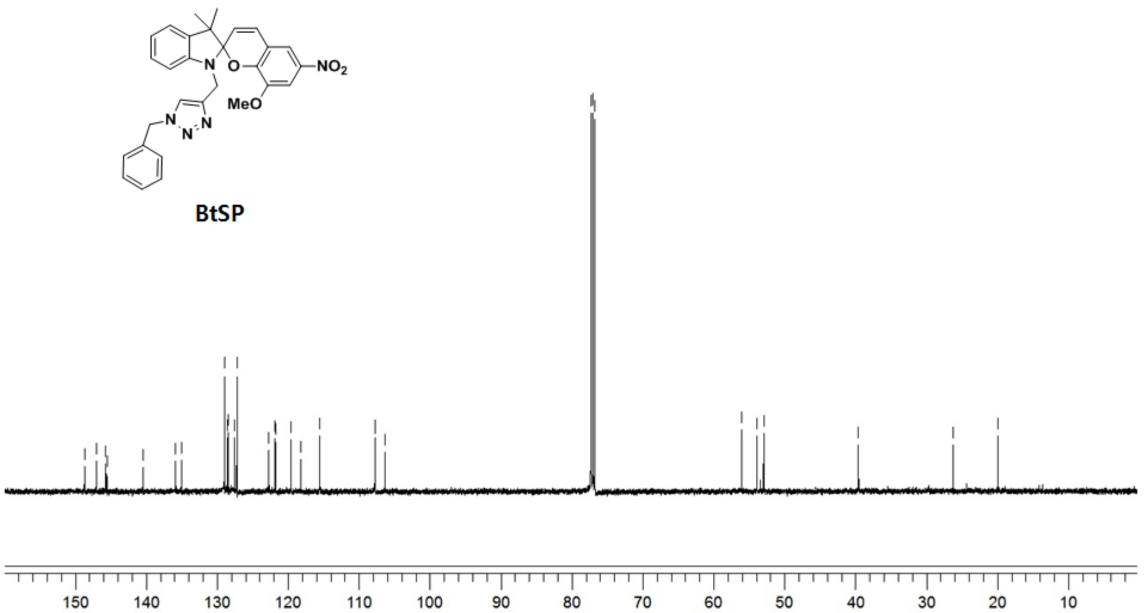

Figure S21. ^{13}C NMR spectrum of **BtSP** (125 MHz, $CDCl_3$).
